# Two-stage video-based convolutional neural networks for adult spinal deformity classification

**DOI:** 10.3389/fnins.2023.1278584

**Published:** 2023-12-11

**Authors:** Kaixu Chen, Tomoyuki Asada, Naoto Ienaga, Kousei Miura, Kotaro Sakashita, Takahiro Sunami, Hideki Kadone, Masashi Yamazaki, Yoshihiro Kuroda

**Affiliations:** ^1^Degree Programs in Systems and Information Engineering, University of Tsukuba, Tsukuba, Japan; ^2^Department of Orthopaedic Surgery, Institute of Medicine, University of Tsukuba, Tsukuba, Japan; ^3^Center for Cybernics Research, University of Tsukuba, Tsukuba, Japan; ^4^Division of Intelligent Interaction Technologies, Institute of Systems and Information Engineering, University of Tsukuba, Tsukuba, Japan

**Keywords:** adult spinal deformity, spinal disorder, video-based method, human action recognition, 3D CNN

## Abstract

**Introduction:**

Assessment of human gait posture can be clinically effective in diagnosing human gait deformities early in life. Currently, two methods—static and dynamic—are used to diagnose adult spinal deformity (ASD) and other spinal disorders. Full-spine lateral standing radiographs are used in the standard static method. However, this is a static assessment of joints in the standing position and does not include information on joint changes when the patient walks. Careful observation of long-distance walking can provide a dynamic assessment that reveals an uncompensated posture; however, this increases the workload of medical practitioners. A three-dimensional (3D) motion system is proposed for the dynamic method. Although the motion system successfully detected dynamic posture changes, access to the facilities was limited. Therefore, a diagnostic approach that is facility-independent, has low practice flow, and does not involve patient contact is required.

**Methods:**

We focused on a video-based method to classify patients with spinal disorders either as ASD, or other forms of ASD. To achieve this goal, we present a video-based two-stage machine-learning method. In the first stage, deep learning methods are used to locate the patient and extract the area where the patient is located. In the second stage, a 3D CNN (convolutional neural network) device is used to capture spatial and temporal information (dynamic motion) from the extracted frames. Disease classification is performed by discerning posture and gait from the extracted frames. Model performance was assessed using the mean accuracy, F1 score, and area under the receiver operating characteristic curve (AUROC), with five-fold cross-validation. We also compared the final results with professional observations.

**Results:**

Our experiments were conducted using a gait video dataset comprising 81 patients. The experimental results indicated that our method is effective for classifying ASD and other spinal disorders. The proposed method achieved a mean accuracy of 0.7553, an F1 score of 0.7063, and an AUROC score of 0.7864. Additionally, ablation experiments indicated the importance of the first stage (detection stage) and transfer learning of our proposed method.

**Discussion:**

The observations from the two doctors were compared using the proposed method. The mean accuracies observed by the two doctors were 0.4815 and 0.5247, with AUROC scores of 0.5185 and 0.5463, respectively. We proved that the proposed method can achieve accurate and reliable medical testing results compared with doctors' observations using videos of 1 s duration. All our code, models, and results are available at https://github.com/ChenKaiXuSan/Walk_Video_PyTorch. The proposed framework provides a potential video-based method for improving the clinical diagnosis for ASD and non-ASD. This framework might, in turn, benefit both patients and clinicians to treat the disease quickly and directly and further reduce facility dependency and data-driven systems.

## 1 Introduction

Spinal diseases and disorders are critical because of their potential to cause major health problems. Spinal diseases have significant physical and functional consequences. They may cause pain, restricted mobility, nerve damage, paralysis, and limitations in activities of daily living. As spinal diseases progress, more severe complications such as chronic pain and loss of neurological function can occur.

Adult spinal deformity (ASD) is characterized by an abnormal spinal shape or posture that results in back or neck pain, physical discomfort, and functional impairments. Different types of ASD require specific treatment approaches. Proper classification helps healthcare professionals design individualized treatment plans. Classification of ASD allows for a better understanding and analysis of different deformity patterns, leading to advancements in treatment and improved patient outcomes. A clear classification facilitates communication among healthcare providers, researchers, and patients, thereby promoting accurate diagnosis, effective treatment, and better patient education. In summary, spinal diseases have serious implications on an individual's health and wellbeing. Classification of ASD is essential for appropriate treatment, research, and effective communication within the medical community.

Currently, in the medical diagnosis of ASD diseases, static and dynamic methods are commonly used. Static methods involve the evaluation of the patient's condition in a stationary or resting position. These methods typically include radiographic imaging techniques, such as X-ray imaging. These imaging modalities provide detailed anatomical information and allow healthcare professionals to assess the structural abnormalities or degenerative changes in the spine and other affected areas. Dynamic methods involve assessing the patient's condition during movements or activities. These methods focus on evaluating the functional aspects of the patient's spine and joints during motion. Examples of dynamic methods include gait analysis, motion capture systems, and electromyography (EMG).

However, some issues remain unresolved. For example, radiographs do not provide information on joint changes when a patient walks. In addition, observing patients' walking increases the workload of medical practitioners. Furthermore, three-dimensional (3D) motion systems are not accessible to all facilities. To solve these problems, an automatic diagnostic system using a conventional RGB camera is proposed in this study. The video can capture joint changes while walking, and the machine-learning method can parse and evaluate the video without the involvement of a doctor, resulting in a reduced workload. In addition, cameras are cheaper and easier to set up than 3D motion systems.

**Figure 1:**
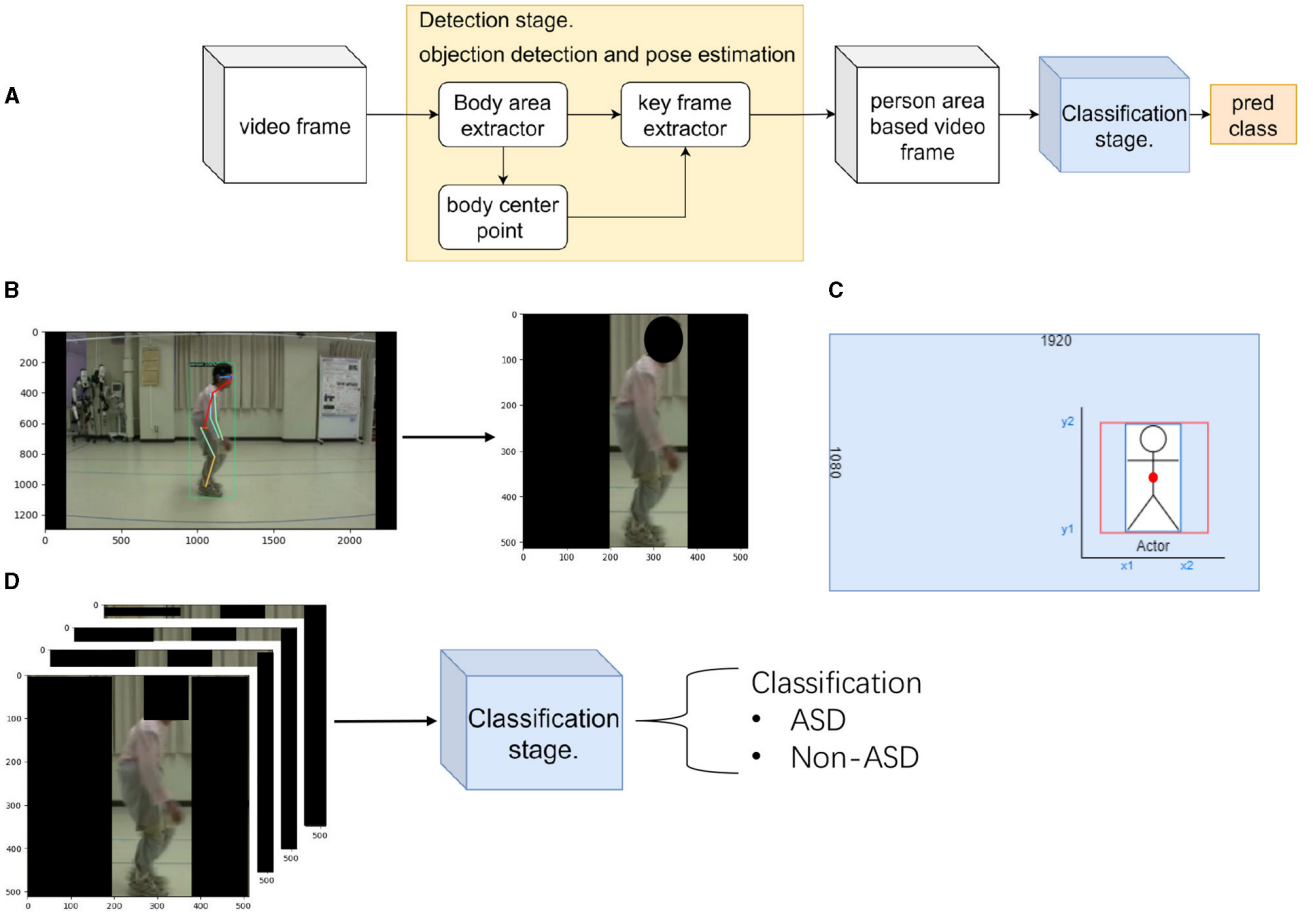
Flow chart of the proposed method. **(A)** shows the flow chart of the proposed method. **(B)** shows the detection stage of the proposed method. Here, we locate the patient and extract their position. **(C)** The calculation method used in the detection stage shows the extraction method for the key frame extractor. **(D)** shows the classification stage of the proposed method. Here, we classify the patient's disease by extracted frames.

In the machine learning field, human action recognition using videos has several advantages: for example, it can reduce the workload, is not limited by the facility, and can capture dynamic motion information from the video (Sun et al., 2020; Zhu et al., 2020). Based on these advantages, our research attempted to apply video-based technology to distinguish between spinal gait diseases. A video-based method for classifying human gait postures that does not interfere with a patient's natural movements was proposed in this study, as illustrated in Figure 1. To the best of our knowledge, this is the first study to evaluate ASD from RGB videos using machine learning techniques.

The contributions of this study are as follows.

- We proposed a two-stage CNN method for human spinal disease diagnosis. We provided a non-invasive and objective method for assessing and analyzing ASD in walking to facilitate diagnosis.
- We identified the importance of detection stage and transfer learning in discriminating ASD and other spinal disorders.
- We compared the predictions of the proposed method to those of two specialized doctors. We proved that the proposed method is effective in classifying ASD.

## 2 Related work

In this section, we present the medical methods used to diagnose ASD and the deep learning methods for human action recognition.

### 2.1 Medical methods

Medical diagnosis of ASD involves the identification and assessment of abnormal spinal curvature and alignment in adult patients. ASD refers to a range of spinal conditions that can cause deformities such as scoliosis (sideways curvature), kyphosis (forward rounding of the upper back), or a combination of both.

Radiographs and quantitative analyses are standard clinical medical methods. Full-spine, lateral standing radiographs are currently the standard for diagnosing ASD and other spinal diseases (Glassman et al., 2005a,b; Lafage et al., 2009; Schwab et al., 2010). However, these are static evaluations of the standing position, which are compensated for by other joints. Careful observation of long-distance walking can provide a dynamic assessment that reveals an uncompensated posture, but it increases the workload of medical practitioners. Quantitative analysis still has limited access in facilities, whereas 3D motion capture systems can successfully detect dynamic changes. Some studies (Miura et al., 2018a, 2020; Severijns et al., 2021; Asada et al., 2023) have reported that dynamic motion systems are useful for the medical diagnosis of ASD.

Severijns et al. (2021) used motion analysis to assess the standing and walking spinopelvic, lower limb kinematics, and lower limb kinetics in a patient's gait. They reassessed ten patients after corrective spinal surgery. Continuous kinematic and kinetic data were analyzed using statistical parametric mapping. A 3D motion analysis was proposed using a Vicon MX system (Miura et al., 2018a, 2020). It provides detailed information regarding the movements of various body segments during the gait cycle. This system typically consists of multiple high-speed cameras placed strategically around a walkway or treadmill, along with specialized software for data collection and analysis. The cameras captured the movements of reflective markers placed on specific anatomical landmarks on the subject's body, such as the pelvis, legs, and feet. This information can be used to assess normal gait patterns, detect abnormalities or deviations from the normal gait, and evaluate the effectiveness of interventions and treatments. Although 3D motion gait systems offer valuable insights into human walking patterns, they have several disadvantages. For example, 3D motion gait systems can be costly to acquire and set up and require specialized equipment such as high-speed cameras, motion capture markers, and dedicated software.

Electromyography (EMG) has been used to investigate the relationship between dynamic and static spinal gait postures (Miura et al., 2018a; Banno et al., 2022). EMG sensor systems are used to measure and record the electrical activity of skeletal muscles. By placing electrodes on specific muscles, researchers can analyze muscle activation patterns and assess the involvement of different muscle groups during walking. The system comprises of 12 EMG sensors and requires them to be placed on the patient's body, generally attached to the trunk and lower limb muscles (Miura et al., 2018a) before each experiment. Banno et al. (2022) compared the postoperative changes in trunk and lower extremity muscle activities between patients with ASD and age-matched controls. Surface EMG was used to measure and analyze muscle activity in both groups. The findings highlighted significant differences in muscle activation patterns, emphasizing the impact of surgical correction on trunk and lower extremity muscle function in patients with ASD. Although the use of EMG sensors in previous studies has provided valuable insights into gait posture recognition, there are disadvantages, such as surface EMG sensors requiring proper skin preparation, including cleaning and shaving, for optimal signal quality.

### 2.2 Deep learning methods

Human action recognition using computer vision is a research field that focuses on the development of algorithms and models to automatically detect and classify human actions in video or image sequences. Its goal is to enable computers to understand and interpret human movements and actions in a manner similar to how humans perceive and recognize them.

There are two common methods for human action recognition: stream-based methods (Simonyan and Zisserman, 2014) and 3D CNN-based methods (Ji et al., 2012). In this study, we focused on 3D CNN methods. A 3D CNN is a deep learning model specifically designed to capture spatiotemporal information from video sequences for human action recognition. Unlike traditional 2D CNNs that operate on individual frames, 3D CNNs consider both spatial and temporal dimensions in their convolutional operations. 3D CNN have been successfully used in a wide range of applications, including video surveillance, sports analysis, and human–computer interaction (Sun et al., 2020; Zhu et al., 2020). In summary, 3D CNN-based methods for human action recognition leverage both spatial and temporal information from video sequences. By considering the temporal dynamics of actions, these methods can effectively capture motion cues and achieve high-performance action recognition.

Research on human-action recognition has aimed to improve the accuracy, robustness, and efficiency of these models. It also explores challenges such as handling variations in lighting conditions, viewpoint changes, occlusions, and complex temporal dependencies between frames. Advancements in deep-learning techniques, particularly the use of convolutional and recurrent neural networks, have improved the state-of-the-art performance of human action recognition tasks. 3D CNN faced problems in this study owing to patient-independent backgrounds, unrelated persons (doctors), and patient gait posture.

However, there is some research about gait classification or recognition, used to diagnose walking disorders such as seen in those with Parkinson's Disease (Kaur et al., 2022), children with cerebral palsy (Dobson et al., 2007), and in post-stroke patients (Kaczmarczyk et al., 2009). In general, gait recognition focuses on the changes in body part alignment during walking (e.g., legs). Kaur et al. (2022) examined the effectiveness of a vision-based framework for multiple sclerosis (MS) and Parkinson's disease (PD). They used pose estimation (OpenPose; Cao et al., 2019) to predict the joint positions of the hip, knee, ankle, and feet to classify MS and PD. The only motivation for our study was attributing gait recognition because of joint changes in the trunk and limbs during walking, as opposed to focusing on the legs alone. ASD is associated with spinal abnormalities. During diagnosis, the doctor will focus more on changes in the spine, but also on changes in the movement of the whole body. The other three patients were diagnosed with ASD. Although they have distinct pathognomonic features, changes in all body movements are important. Because we cannot limit the features of a disease to a single point (e.g., the leg), we used human action recognition to learn how the features of the entire body changed during walking without limiting it to a single body part. This is also true of some medical studies (Miura et al., 2018b, 2020) that have diagnosed these diseases. They used a motion system to dynamically capture changes in the joints throughout the patient's body as they walked. They focused on changes throughout the patient's body rather than on changes in a specified part. For these reasons, we used human action recognition to analyze the changes in a patient's entire body during walking.

In summary, we created a comparative table of the current medical diagnostic methods for ASD and other spinal disorders, as well as their differences with deep learning methods for human action recognition, as shown in Table 1.

**Table 1:** Comparative table about the medical methods and deep learning methods.

**Method**	**Diagnostic process**	**Contact with the patient's body**	**Physician review required**	**Facility dependency**
**Medical diagnosis methods**				
Quantitative analyses	Patient fills out the paperwork and the doctor makes a diagnosis based on the paperwork	Yes	Yes	Questionnaires
Radiographs	Patient are diagnosed with radiographs beforehand after judged by a doctor.	No	Yes	X-ray machine
Dynamic methods	Sensors need to be placed on the patient's body and doctor makes a diagnosis based on the capture results	Yes	Yes	Dynamic capture system
**Deep learning methods (Video based)**				
Two-stream method	Recorded video beforehand, and need to predict Optical Flow in advance	No	No	Camera
3D CNN method (Ours)	Using one camera to recorded the patient. More concerned with characteristics of the whole body.	No	No	Camera
Kaur et al. (2022)	Using two cameras to recorded the patient, then pre-processed (pose estimate, etc.), and classification. More attention to partial features (lower extremities).	No	No	Camera

## 3 Methods

### 3.1 Proposed two-stage method

In this section, we describe the proposed two-stage method for distinguishing ASD from other spinal disorders. Figure 1 shows an overview of the proposed method. Figure 1A is a flow chart of the proposed method. There are two stages: the preprocessing method (**detection stage**) and motion classification network (**classification stage**). The first stage separated the patient's area from the background of the image. In the second stage, the extracted patient area was used to distinguish the gait of spinal posture. Several evaluation metrics are used in the experiments.

#### 3.1.1 Detection stage

In our dataset, the video recording of a walking patient was performed using a single RGB camera fixed to one side. The patient walked in front of the fixed camera. There were two difficulties at this stage. First, the videos contained irrelevant information other than the patient's body, such as tables, fans, whiteboards, or curtains, other than the patient's. Second, some patients required a doctor to walk with them. This leads to scenes in which the doctor is present within a certain frame. However, it is difficult to standardize these conditions at different medical institutions.

In this stage, using a preprocessing method, the first problem was solved using a **body-area extractor**. The second problem can be solved by extracting the bounding box in which the patient walks with the doctor using a **key-frame extractor**. In order to obtain the patient's center point, we ensured that the extraction screen was always at the center of the patient. Figure 1B illustrates the detection stage method. At this stage, the patient area has been extracted from the background image.

**Body area extractor** separates the patient's body and background information. We used a Faster RCNN (Ren et al., 2015) pretrained on the COCO dataset, which demonstrated effective detection results for various tasks. We applied detection based on the Faster RCNN for each frame and adjusted the extracted area to 512 × 512 pixels to facilitate the calculation.

**Body center point** uses the bounding box detected by **Body area extractor**. To ensure the accuracy of the calculation using the detected bounding box, we simultaneously predicted the keypoints of human pose. A Faster RCNN framework can easily be extended to human pose estimation (Ren et al., 2015). We used the COCO pose dataset, which provides 17 keypoints for human figures. We compared the two predicted hip joint center points (left and right hips) with the center point calculated from the bounding box. In Figure 1B (left), it shows the predicted joint keypoint in frame. This step ensures that the patient is always in the middle of the extraction area, and the calculated center point is used in the next step (**key frame extractor**).

**Key frame extractor** attempts to differentiate between the patient and another person. In our dataset, we observed that the patient always entered the frame before the doctor. Therefore, doctors always follow their patients. Using this information, **key-frame extraction** was performed in three steps. First, object detection was applied to the first frame of the video to determine the bounding box of the patient. Second, a bounding box was used to determine the center point of the patient's body. Third, we recorded the body center point coordinates in the first frame and compared them with those in the subsequent frame. The same person must move the shortest distance between the two adjacent frames. The coordinates of the patient's locations were obtained following the three steps. This determines whether the patient is the only person in the extracted area. Although the detection returned a rectangular area, the CNN predicted a square region. Figure 1C shows the calculation of the extraction method. To ensure the aspect ratio of the characters, we use the retained height of the patient (y2 and y1 coordinates of the bounding box) as the base length for cropping. Both sides of the extracted areas were filled to ensure that the images were not distorted. Therefore, an extracted area box (red box) was obtained from a specified bounding box (blue box). Finally, all video frames were resized to 512 pixels to achieve 30 FPS.

**Figure 2:**
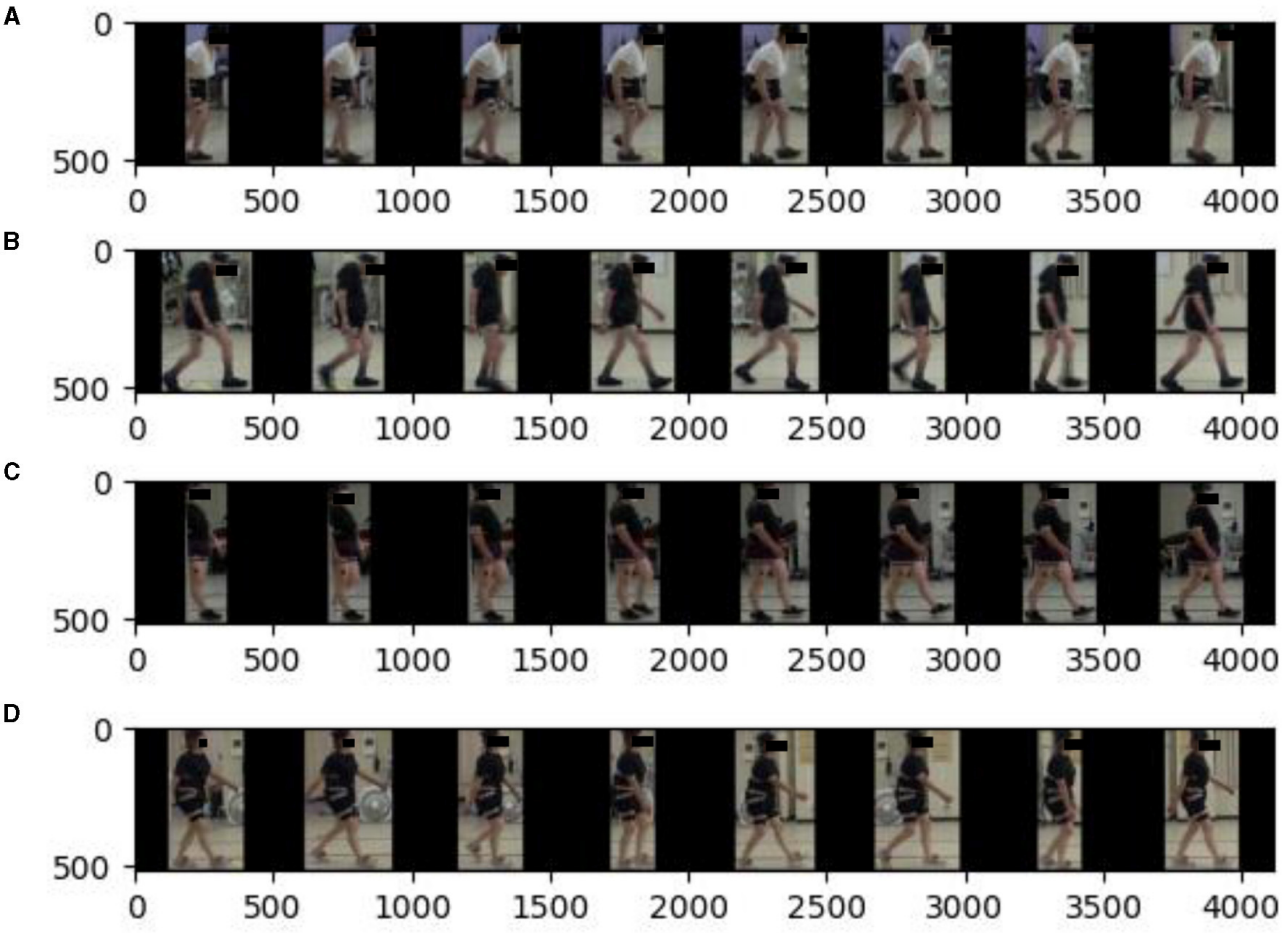
Extracted results from detection stage. Uniformly extracted eight frames from 1 s for train or validation. We mosaiced the patients' faces to protect theri privacy. There was no mosaic during training or validation. **(A)** ASD. **(B)** DHS. **(C)** LCS. **(D)** HipOA.

The results obtained after the detection stage are presented in Figure 2. The four pathologies, ASD, DHS, LCS, and HipOA are listed here. Each row consisted of eight frames uniformly extracted from a 1 s video. We used the data obtained for model training (see the next section for details). The radiographic spinal parameters during the gait analysis are shown in Table 2. Each patient was asked to stand comfortably. Static spinal parameters were evaluated as follows: sagittal vertical axis (SVA); thoracic kyphosis (TK); lumbar lordosis (LL); pelvic tilt (PT); pelvic incidence (PI); TI pelvic angle (TPA); coronal Cobb angle of the thoracolumbar and lumbar scoliosis (CObb). The surgeon based the diagnosis of ASD and non-ASD on these spinal parameters and clinical information, including the chief complaints.

**Table 2:** Radiography parameters for ASD and other spinal disorders, e.g., SVA, sagittal vertical axis; TK, thoracic kyphosis; LL, lumbar lordosis; PT, pelvic tilt; PI, pelvic incidence; TPA, T1 pelvic angle.

	**ASD**		**non-ASD**	
**Parameter**	**Mean**	**Range**	**Mean**	**Range**
Age (years)	70	40 to 84	70	22 to 81
Height (cm)	150	131 to 172	152	134 to 175
SVA (mm)	128.0	−11 to 238	10.95	−97 to 155
TK (°)	21.52	−23 to 67	14.03	−61 to 55
LL (°)	8.956	−33 to 49	36.03	−7.3 to 63
PT (°)	33.66	0.2 to 63	28.43	6 to 61
PI (°)	48.97	18 to 76	49.73	25.6 to 74
TPA (°)	39.00	0.8 to 71	27.04	−7.4 to 46.7

#### 3.1.2 Classification stage

Figure 1D illustrates the second stage. This stage uses the extracted patient area to classify the spinal diseases. We constructed a motion classification network based on a ResNet-style (He et al., 2015) 3D CNN with a bottleneck block. The detailed structure is given in Table 3. The motion network receives a series of frames extracted from the detection stage, indicating that only the patient area is used.

**Table 3:** ResNet3D architectures considered in our experiments.

**Layer name**	**Output size**	**ResNet3D-bottleneck**
conv1	T × 112 × 112	3 × 7 × 7, 64, stride 1 × 2 × 2
pool1	T × 56 × 56	max, 1 × 3 × 3, stride 1 × 2 × 2
conv2_x	T × 56 × 56	$\left[\begin{array}{c} 1\times 1\times 1,256\\ 3\times 3\times 3,64\\ 1\times 1\times 1,256 \end{array}\right]\times b_{1}$
conv3_x	T × 28 × 28	$\left[\begin{array}{c} 1\times 1\times 1,512\\ 3\times 3\times 3,128\\ 1\times 1\times 1,512 \end{array}\right]\times b_{2}$
conv4_x	T × 14 × 14	$\left[\begin{array}{c} 1\times 1\times 1,1024\\ 3\times 3\times 3,256\\ 1\times 1\times 1,1024 \end{array}\right]\times b_{3}$
conv5_x	T × 7 × 7	$\left[\begin{array}{c} 1\times 1\times 1,2048\\ 3\times 3\times 3,512\\ 1\times 1\times 1,2048 \end{array}\right]\times b_{4}$
pool5	1 × 1 × 1	spatiotemporal avg pool, fc layer with sigmoid

Convolutional residual blocks are shown in brackets, next to the number of times each block is repeated in the stack.

In Table 3, the dimensions of the filters and outputs are time, height, and width. The *T* of the study was selected as eight, meaning that eight frames were uniformly extracted from the 1 s video. *b*_1_, …, *b*_4_ are the number of blocks implemented at conv2_x, conv3_x, conv4_x, conv5_x (defined 3, 4, 6, and 3, respectively). This means, for example, that *b*_1_ was repeated 3 times. The series of convolutions culminates in a global spatiotemporal pooling layer that yields a 2,048-dimensional feature vector. This vector is fed into a fully connected layer that gives outputs of the class probabilities through a sigmoid.

ResNet solves the CNN degradation problem. This allows the network to learn more features, resulting in a higher performance. Some state-of-the-art 3D CNN structures also borrow the ResNet structure to improve video recognition ability (Kay et al., 2017; Sun et al., 2020; Zhu et al., 2020). In this study, we trained a ResNet-style 3D CNN using videos to recognize a patient's gait posture. Similar to Ji et al. (2012), we extracted eight frames uniformly from the 30 image frames for training.

#### 3.1.3 Evaluation metrics

We compared the predictions of our model with those of doctors using three different evaluation metrics. The metrics used were mean accuracy, F1 score, and area under the receiver operating characteristic curve (AUROC). All evaluation metrics were averaged across five-fold cross-validation.

Equation (1). *y* is the true label and ŷ is the predicted class label. A higher score indicated that the model had a greater ability to classify positive (ASD) and negative (non-ASD) labels.


(1) $$Accuracy=\frac{1}{N}\sum_{i}^{N}1\left(y_{i}=\hat{y_{i}}\right)$$

For the F-1 score, refer to Equation (2). The F1 score is calculated based on the precision and recall of the validation data. where the precision is the number of true-positive (ASD) results divided by the total number of positive results. The recall is the number of true-positive results divided by the total number of samples that should have been identified as positive. The model exhibited perfect precision and recall with a high F-score.


(2) $$\begin{array}{l} F1=2\frac{precision*recall}{precision+recall} \end{array}$$

The AUROC score summarizes the ROC curve as a single number that simultaneously describes the performance of the model for multiple thresholds. This ensured the model's ability to classify a given task. An AUROC score of 1 is an ideal score, and an AUROC score of 0.5 corresponds to random guessing.

**Figure 3:**
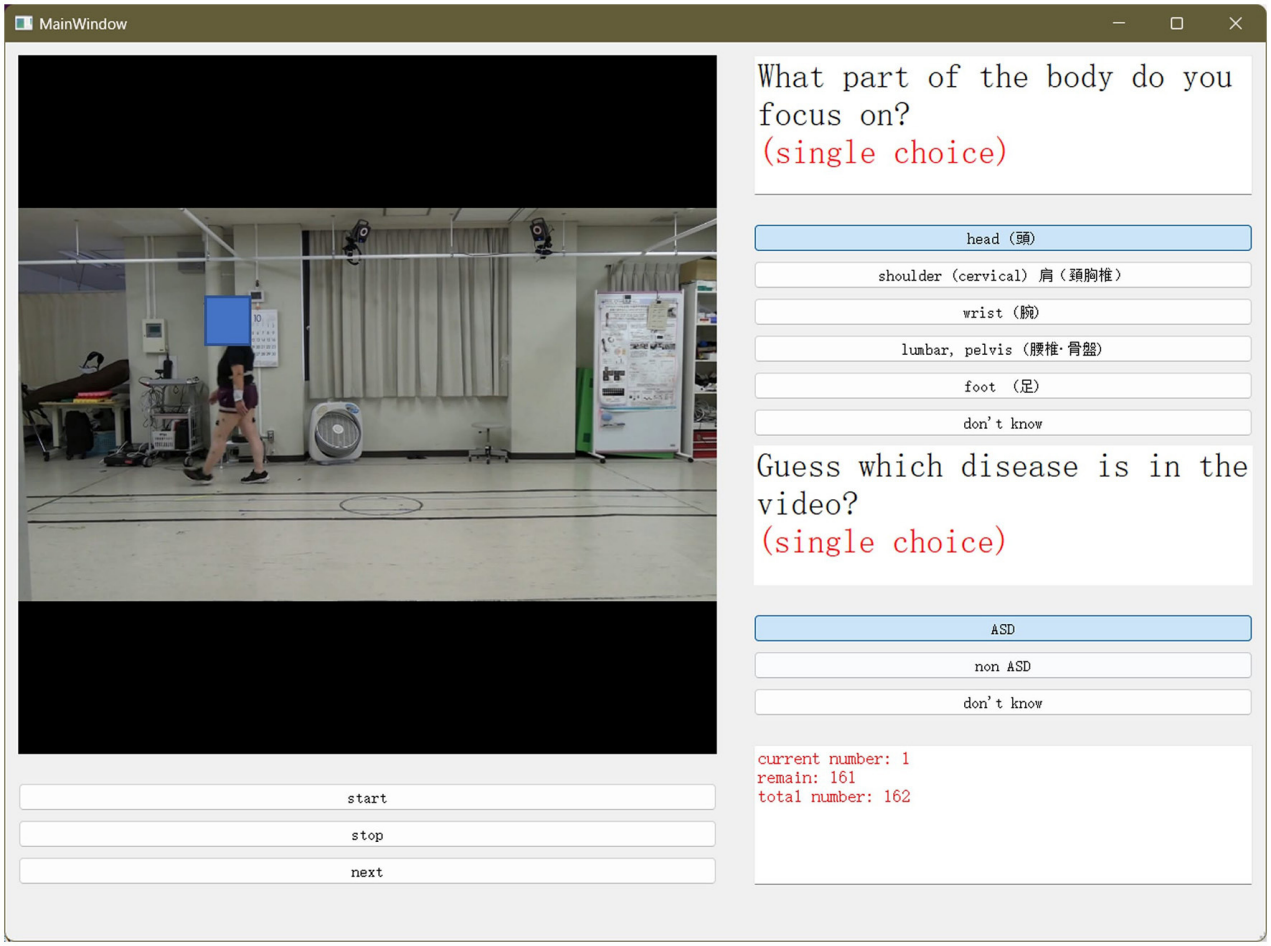
Graphical user interface (GUI) used for the doctor's experiment. Two specialized doctors were asked to select what they considered the classification of the disease, and the body part to be noted. This was performed by watching the videos.

To conduct a doctor observation experiment, we developed a graphical user interface (GUI) for doctors. On the screen in Figure 3, the doctor selects the spinal disease and focuses on the part of the analysis when the patient walks. We displayed 81 clips of patients' movements at the beginning and end, each containing a video 1 s long. Two specialist doctors were asked to select what they considered the classification of the disease and the body parts to be noted by watching the videos. A period of 1 s was chosen as it is comparable to the time required for a patient to enter a small examination room in a clinic and walk toward the doctor.

We counted the results of doctors' choices and compared them with those of the proposed model. In Section 4, we compare the relationship between the predictions of the proposed model and those of the doctors.

## 4 Experimental results

In this section, we first describe the gait video dataset used in this study and then introduce the experimental settings. Subsequently, we compare our method with those used in traditional studies on two-stream networks (Simonyan and Zisserman, 2014) and the results obtained by the two doctors. Ablation experiments were conducted to demonstrate the effectiveness of the detection-stage method and transfer learning. We also present the model visualization results to explore where the model's concerns lie.

### 4.1 Gait video dataset

In this section, we describe the gait video dataset. To advance the research on gait spinal posture in ASD and other spinal diseases for medical analysis, we collected videos used in Miura et al. (2018a), Miura et al. (2018b, 2020), and Asada et al. (2022) and organized them into datasets. This study was approved by the Ethics Committee of the University of Tsukuba Hospital (H30-087). This study was performed in accordance with the contemporary amendments to the Declaration of Helsinki and within an appropriate ethical framework. This dataset consists of video recordings of individuals with ASD and a control group capturing their walking patterns and spinal postures. This dataset aims to provide valuable insights into the relationship between gait abnormalities and spinal postures. The collection of this gait posture video dataset involved recruiting participants from ASD clinics or research centers and obtaining informed consent. Participants were instructed to walk naturally while being recorded using high-quality video cameras at a fixed angle.

**Figure 4:**
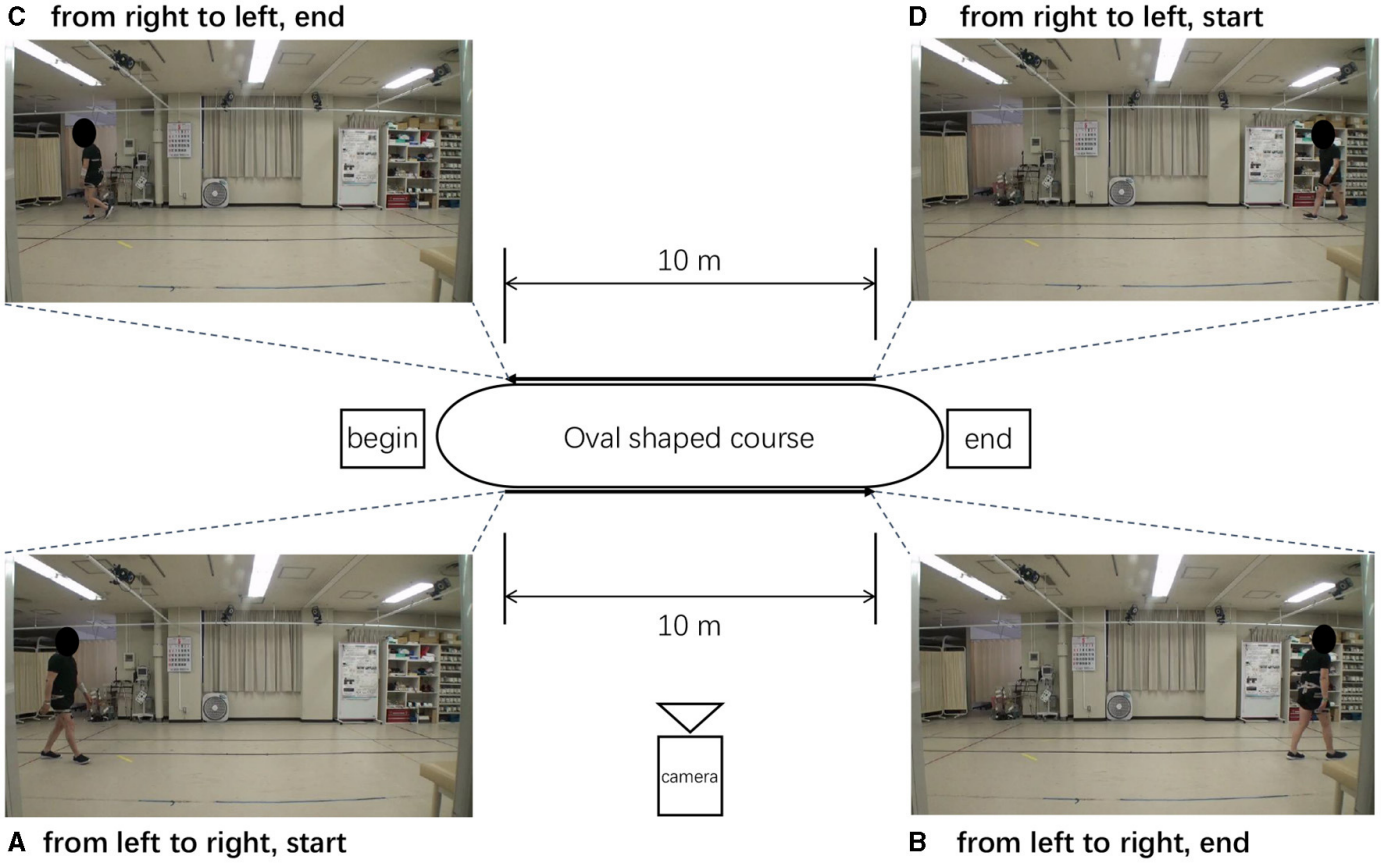
Oval-shaped course used when recording the walking. **(A–D)** Different stages of patient walking.

Figure 4 shows an oval-shaped course used for walking in an indoor space at a distance of 10 m. patient's walked from left to right (or from right to left), and the patient's walking posture was recorded from a side view with the camera set at a fixed location. Each video was captured at a frame rate of 30 FPS (frames per second) and a resolution of 1920 × 1080 HD, and the video quality was set to high. The video format used was MP4 and the codec was H.264. The patients were asked to record a video from the start to the end-which is when they were unable to walking.

The original videos lasted 60–300 s. Consecutive frames from each original video were clipped to avoid scenes without individuals within the frame. The sliced video contained 1,957 individual video clips ranging from 2s to 10s. The gait posture's label attached to each video was based on the spine surgeon's diagnosis from diagnostic radiographical assessment using standing whole-spine X-ray images and the clinical symptoms of the patients. Table 4 presents the detailed information regarding our dataset. The dataset comprised videos of 81 patients (61 males and 20 females). Their ages ranged from 22 to 84 years (mean: 70 years). Details of the dataset are shown in Table 4.

**Table 4:** Dataset details.

**Label**	**Abbreviations**	**Diseases detail name**	**Patient**	**Clipped video**	**Total**
ASD	ASD	Adult Spinal Deformity	54	1,046	1,046
	DHS	Dropped Head Syndrome	16	587
non-ASD	LCS	Lumbar Canal Stenosis	9	260	911
	HipOA	Hip Osteoarthritis	2	64	

Our facility performs gait analysis for various musculoskeletal pathologies, including ASD (adult spinal deformity (ASD), LCS (lumbar canal stenosis), DHS (dropped head syndrome), and HipOA (hip osteoarthritis). This study aimed to distinguish between patients with ASD from those with other spinal disorders. Owing to the difficulty in diagnosing ASD, we focused on classifying ASD using gait analysis due to the difficulty of diagnosis of ASD. The diagnosis of ASD requires comprehensive clinical judgment based on the clinical symptoms of gait problems as well as radiographic assessment. This makes it difficult to easily diagnose ASD, which can take time and cause delays in diagnosis. Therefore, the present study is of significant interest. Another reason for this is data imbalance. The ASD sample size of 54 patients was considerably larger than that of patients with other diseases (DHS 16, LCS 9, and HipOA 2). In such cases, training a model to classify all four diseases separately can lead to biased predictions and poor performance for minority classes. Therefore, we classified ASD as ASD label and the other three diseases (DHS, LCS, and HipOA) as non-ASD.

#### 4.1.1 Five-fold stratified group cross-validation

In general, machine learning divides a dataset into three parts: train/val/test dataset. However, because of our relatively small dataset (Table 4), we did not divide the entire dataset into separate sets. Instead, we use five-fold cross-validation to obtain stable and generalizable results (Refaeilzadeh et al., 2009).

**Figure 5:**
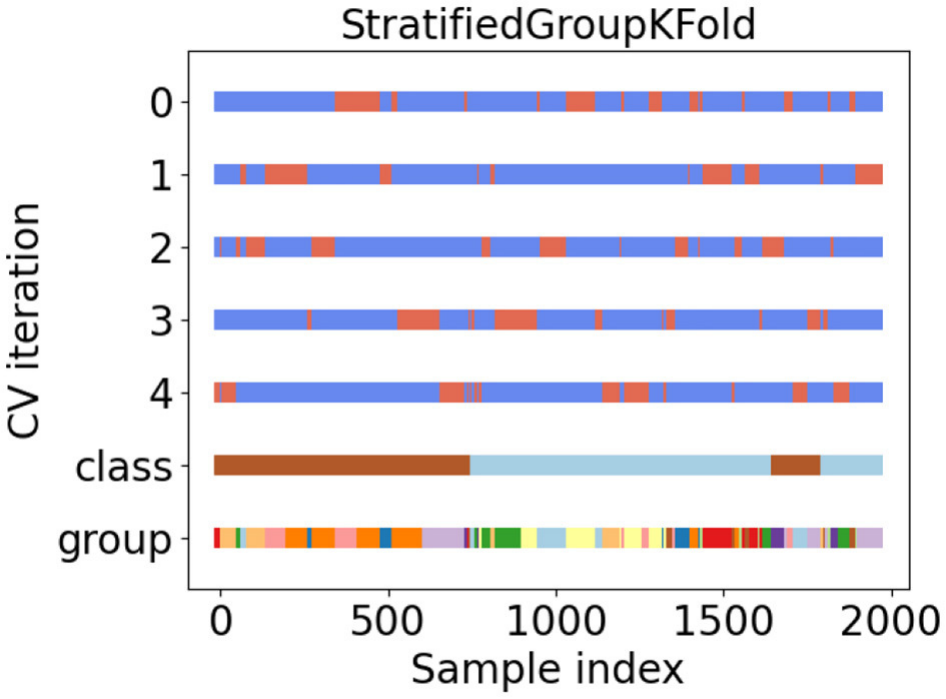
Five-fold stratified group cross validation for our gait video dataset.

During the experiment, we ensured that the same patient did not appear simultaneously in the training and validation datasets at the same time. In addition, the balance of patients among the folds was a significant problem. In this study, we used a method called stratified group cross-validation. This ensured that the number of patients was balanced and that the video of the same patient did not repeat in the training and validation datasets. Figure 5 shows the data distribution after five-fold stratified group cross-validation. We used the API from the scikit-learn toolkit (Pedregosa et al., 2011) and ensured a balanced dataset for our experiment.

In Figure 5, tags 0 to 4 represent five different dataset classifications. They divided the datasets into training and validation sets. Blue represents the training set, and orange indicates the validation set. Class tags represent the overall data: light blue for ASD patients and brown for non-ASD patients. Different colors in the group tag represent different independent patients. There were 81 different colors (patients).

### 4.2 Experiments

#### 4.2.1 Training

Training in the classification stage was performed using ResNet-50 style (He et al., 2015) 3D CNNs along with the bottleneck block. A pretrained model was trained on the Kinetics-400 (Kay et al., 2017) dataset. We split the video clips into 1 second shots, including 30 frames. Subsequently, we uniformly extracted eight frames from the 30 frames for training, as described in Kay et al. (2017). We trained our models using a single A5000 GPU with 24 GB of memory and the PyTorch framework 1.13.1 version. The batch size for training was set to 8. We used an early stop technique to speed up the training. When the validation data prediction loss did not increase within five epochs, the training ended and the training checkpoint was saved. The entire motion-classification network was trained on our walking dataset using the Adam (Kingma and Ba, 2014) optimizer, with a learning rate of 10^−5^. The learning rate is reduced by half if the validation accuracy does not increase during the three epochs.

#### 4.2.2 Testing

We report the mean accuracy, mean F1 score, and AUROC across a five-fold cross-validation of the validation dataset. To ensure fairness during testing, our test frame was set to eight, which is the same as that of the training frame. We also compared the predictions of the model with those of two specialized physicians.

To date, video-based studies of disease diagnostic associations between ASD and non-ASD do not exist to the best of our knowledge. Therefore, a more detailed comparison was required. Nevertheless, we compared two more common approaches to human action recognition. In the first video-based study of ASD and non-ASD, we focused more on the predictive results of the proposed model compared to the results derived from physician observation videos. The two-stream method was compared with the proposed method. In the two-stream method, videos are clipped into independent frames. To capture complementary information on the appearance of still frames and the motion between frames, the two-stream method uses the optical flow (OF) technique to estimate the motion change in three frames. We refer to the original paper structure, where a ResNet50 structure pretrained on the ImageNet dataset (Deng et al., 2009) was used to extract the features from a single frame, predict the OF, and then fuse their predicted scores to obtain the final prediction results. We also compared the results of the two physicians' observational experiments. For the results predicted by the doctors, we present the results for each doctor after counting. Both doctors watched the same videos during the experiment.

### 4.3 Results

Table 5 lists the results for the different models. The metrics included the mean accuracy, F1 score, and AUROC of the different methods. For doctors 1, the metrics were 0.4815, 0.4474, and 0.5185, respectively. For Doctor 2, the metrics were 0.5247, 0.4615, and 0.5463, respectively. The metrics for the two-stream method were 0.7063, 0.6085, and 0.7797. The metrics for the proposed method were 0.7553, 0.7063, and 0.7864. The proposed method exhibits better metrics than the two-stream and two-doctor observation methods.

**Table 5:** Mean accuracy values, F1 score, and AUROC (area under the ROC curve) for our experiments.

**Models**	**Mean accuracy**	**F1 score**	**AUROC**
Doctor 1 results	0.4815	0.4474	0.5185
Doctor 2 results	0.5247	0.4615	0.5463
Two-stream method	0.7063	0.6085	0.7797
Two stage method (ours)	**0.7553**	**0.7063**	**0.7864**

Values in bold indicate the highest value compared to other methods.

**Figure 6:**
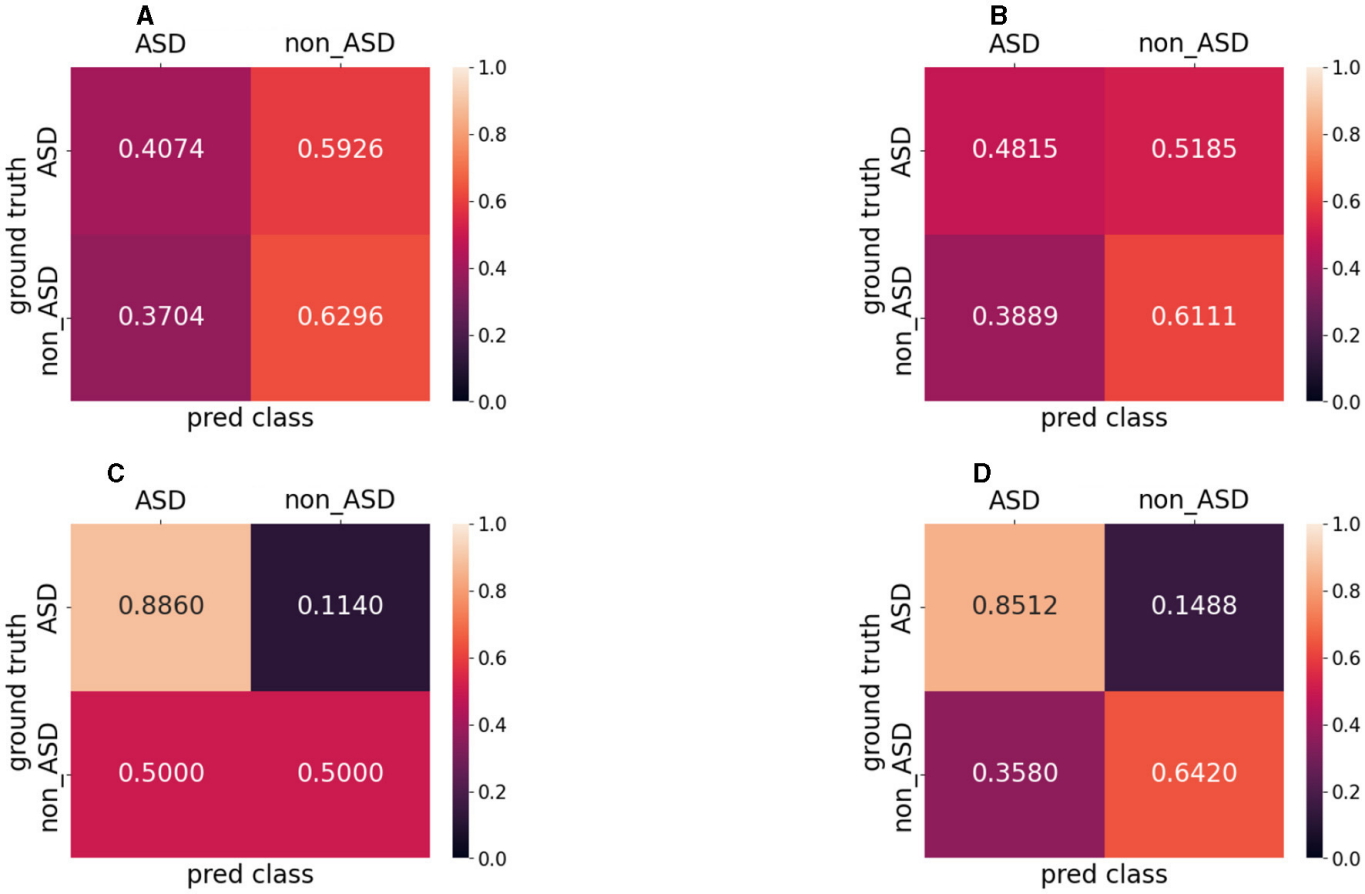
Confusion matrix of different experiments. Counting the five-fold cross-validation results. **(A)** Doctor 1 result. **(B)** Doctor 2 result. **(C)** Two-stream method. **(D)** Proposed method.

Figure 6 presents the confusion matrix for the proposed method. Counting the five-fold cross-validation results, ASD has 1,046 video clips and non-ASD has 911 video clips. Our dataset contained 1,957 video clips, as described above. Lighter colors indicate a higher ratio of video clips among the target clips, while darker colors indicate a lower ratio of video clips among the target clips. The first and second rows represent videos labeled by the doctor (ground truth), and the first and second columns represent the results predicted by the model. We define the first row of the first column as a true positive (TP), the first row of the second column as a false positive (FP), the second row of the first column as a false negative (FN), and the second row of the second column as a true negative (TN). For presentation purposes, the numbers in the graph represent the ratios normalized to the targets.

Doctors 1 and 2 both had a poor ability to distinguish ASD, with 0.4074 TP and 0.5926 FP for doctor#1 and 0.4815 TP and 0.5185 FP for doctor#2. However, they could distinguish non-ASD, with 0.3704 FN and 0.6296 TN for Doctor #1, and 0.3889 FN and 0.6111 TN for Doctor #2. It is difficult to make a correct diagnosis from very short videos (1 s with eight frames), even for experienced doctors. For the two-stream method, TP is 0.8860 and FP is 0.1140, respectively. TN and FN were equal at 0.5000. For the proposed method, TP and TN were 0.8512 and 0.6420, respectively. The FP is 0.1488, and the FN is 0.3580, respectively.

### 4.4 Ablation study

Ablation experiments were conducted to demonstrate the effectiveness of the proposed method. Specifically, we investigated the usefulness of the preprocessing method (detection stage) and compared its effectiveness with and without transfer learning. Table 6 lists the mean accuracies of different experiments.

**Table 6:** Mean accuracy values for the ablation study.

**Models**	**w Preprocessing method (detection stage)**	**w/o Preprocessing method (detection stage)**
Train from scratch	0.6167	0.5134
Transfer learning	**0.7553**	0.4597

We test the mean accuracy with the detection stage method and transfer learning. Values in bold indicate the highest value compared to other methods.

For the video without preprocessing, because a square frame was necessary, we used a short-sided scale (Tran et al., 2014; Kay et al., 2017) to crop the 1920 × 1080 size frame to a 512 × 512 size to match our proposed method. The short-side scale determines the shortest spatial size of the video (i.e., width or height) and scales it to a given size. The longer side was scaled to maintain the aspect ratio. Our transfer learning method is based on a model pre-trained on the Kinetics-400 dataset (Kay et al., 2017) and finetuned on our dataset.

For training the Kinetics-400 dataset, we selected the method reported by Kay et al. (2017). We used the same sampling method (Section 4.2) to uniformly extract eight frames from a 1 s video slice. The final pre-trained metrics were 74.58 (Top-1 ACC) and 91.63 (Top-5 ACC). As the Kinetics-400 dataset has 400 different categories, we used only two categories in our dataset. Therefore, to fine-tune the pre-trained model, we modified the last layer from 400 to 2 to match our dataset categories. The entire model was then retrained. We ensured that the learning rate, optimizer, and early stop were consistent in both the pre-train and fine-tune phases. Detailed information is provided in Section 4.2.

Moreover, to determine the effect of a patient's postural changes throughout the walking process on the final outcome, we divided the entire video dataset of the patient walking into three parts: start, middle, and end. They walked through the start, middle, and end points. Training was performed using the entire dataset, and testing was performed using a split dataset. For testing, the number of videos in the split dataset was one-third that of the entire dataset, but the distribution of the five-fold cross-validation remained consistent with that of the overall dataset.

**Figure 7:**
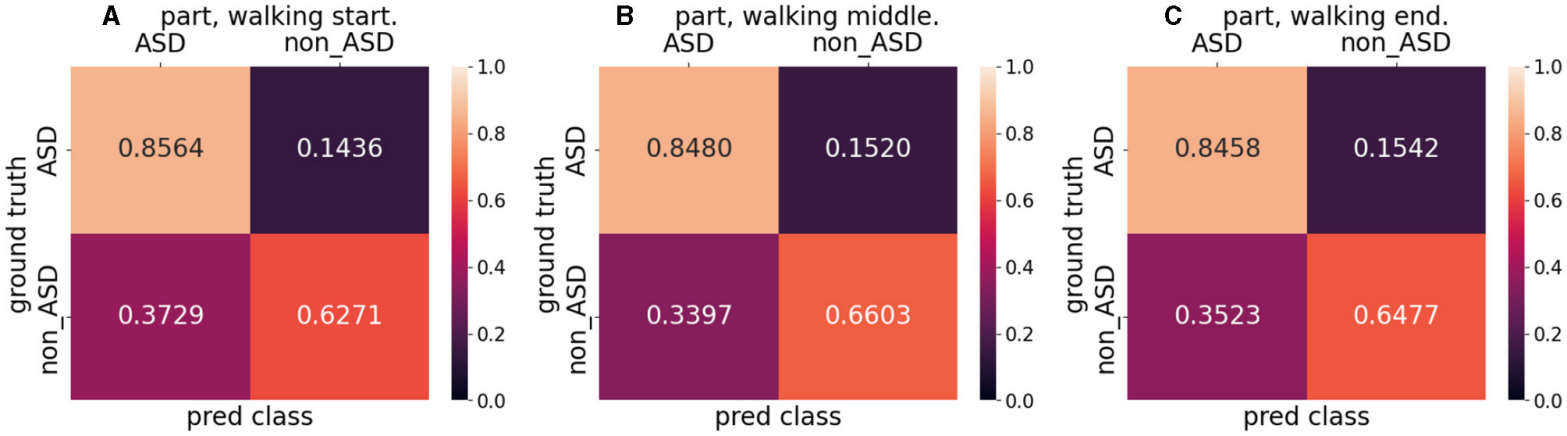
Confusion matrix for split dataset. **(A)** indicates the walking start part, **(B)** indicates the walking middle part, and **(C)** indicates the walking end part.

Table 7 lists the mean accuracy, F1 score, and AUROC for the split dataset test results. Parts B and C had a higher mean accuracy (0.7600 and 0.7582, respectively) than the mean accuracy (0.7559), whereas Part A had a lower accuracy (0.7495). The F1 scores for parts A and C were almost equal (0.7000 and 0.7032, respectively) but lower than those for part B (0.7206). The three parts of AUROC agree roughly. This was almost identical to the entire dataset's results when comparing the mean accuracy (0.7559), F1 score (0.7079), and AUROC (0.7867). Figure 7 shows the confusion matrix for the split dataset. The TP values for the different parts are almost equal, but the FN values for parts B and C are higher than that for part A. This means that when the walking time increased, the accuracy of non-ASD discrimination increased slightly.

**Table 7:** Mean accuracy values, F1 score, and AUROC for the split dataset.

**Models**	**Mean accuracy**	**F1 score**	**AUROC**
A part	0.7495	0.7000	0.7774
B part	0.7600	0.7206	0.7941
C part	0.7582	0.7032	0.7886
Mean	0.7559	0.7079	0.7867

Our dataset is divided into three parts: A represents the walking start, B represents the walking middle, and C represents the walking end.

### 4.5 Model visualization

This study presents a model visualization of the results of the proposed method. Model visualization can help us understand which part of the model is focused on during training. In addition, determining the part of the patient's body that is involved in walking provides more positive information. Grad CAM and Grad CAM++ (Chattopadhyay et al., 2017; Selvaraju et al., 2017) are techniques used to explain decisions visually using CNN-based models. Grad CAM++ is superior in terms of object localization, and explains multiple object instances in a single image. In this study, we used the code from Gildenblat et al. (2021) to visualize the model results.

**Figure 8:**
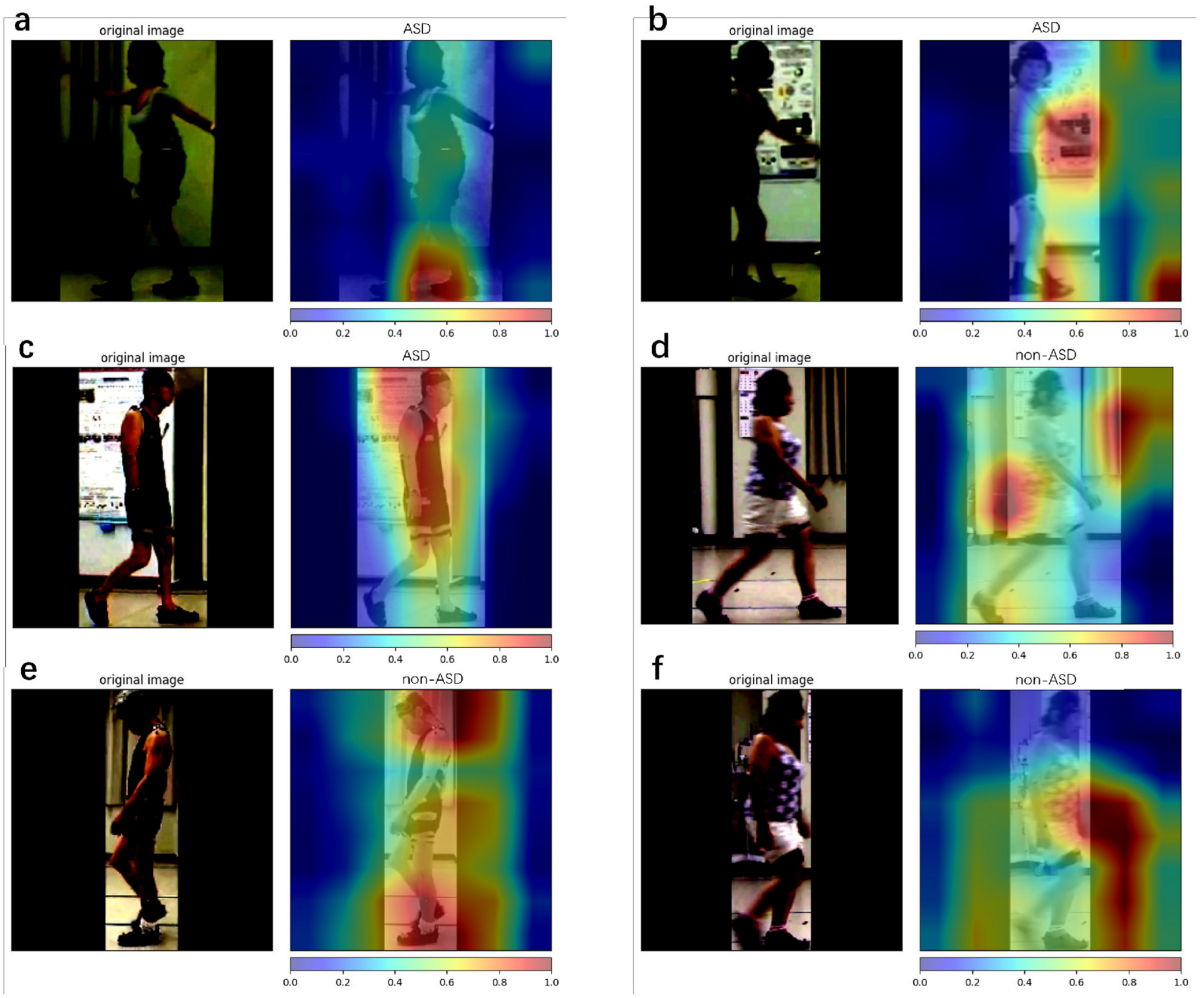
Class attention map (CAM) is used for the proposed method. Here, we use Grad CAM++ to visualize our model prediction. Red represent areas that provide more positive information about the final result, and blue represent areas that provide less positive information about the final result. **(a–c)** The results with ASD patients and **(d–f)** the results with non-ASD patients. **(a, c, e)** Denote the successful cases, while **(b, d, f)** denote the failure cases.

Model visualization cannot represent temporal information. However, We calculated attention maps at different frames and fused them into a single image for visualization. In each frame, we attempted to reflect the relevant information. Figure 8 shows the model visualization results obtained using the proposed method. The correctly classified samples (Figures 8A, C, E) are shown on the left and the incorrectly classified samples (Figures 8B, D, F) are shown on the right.

## 5 Discussions

This study presents a two-stage method for diagnosing ASD and other spinal gait diseases in a video format. The proposed method was compared to the traditional two-stream method, and two doctors observed the video results. The proposed method is a convenient, low-facility-dependent, fast, and accurate remote diagnostic tool for ASD and other diseases. Our framework is open-source and available at https://github.com/ChenKaiXuSan/Walk_Video_PyTorch. We have provided an organized experimental code to simplify the repetition of our experiments.

Based on the results presented in Table 5, we found that our proposed two-stage method had a higher mean accuracy than the results obtained by two different specialized doctors. The mean accuracies of the two doctors were 0.4815 and 0.5217, respectively. For the binary classification task, an accuracy of 0.5 indicates that the disease could not be correctly discriminated from the short video. This proves that it is difficult, even for experienced doctors, to discriminate between ASD and other spinal gait diseases through the observation of 1 s videos. A traditional two-stream method that outperformed the results obtained by the two doctors was also tested in this study. The OF used in the two-stream method can extract information between different frames more accurately than humans (e.g., the movement of pixel positions by a patient's gait). The mean accuracy score predicted by the proposed method was the highest at 0.7553.

The accuracy metric is reliable only if the dataset is class balanced, whereas our dataset is class imbalanced. The F1 score considers both precision and recall, making it an appropriate metric for evaluating the overall performance of a model. Thus, we selected the F1 score in conjunction with the accuracy as the standard for evaluating the proposed method. A higher F1 score indicates that the model effectively identified both positive (ASD) and negative (non-ASD) cases with high accuracy. An F1 score of 0.7063 for the proposed method indicates that it performed well in correctly identifying both ASD and non-ASD cases, making it a more reliable evaluation metric for class-imbalanced datasets. This shows that the proposed method is capable of distinguishing between patients with ASD and non-ASD more accurately than the other methods and exhibits a better overall classification ability.

AUROC is a valuable metric for evaluating the performance of binary classification models, particularly for imbalanced datasets. This indicates how well the model can distinguish between positive (ASD) and negative (non-ASD) classes. A high AUROC is essential in medical diagnostic tasks because it ensures that the model can reliably differentiate between patients with and without the disease. The proposed method exhibits the highest AUROC (0.7864). This indicates that the proposed method can distinguish between patients with ASD and those without ASD. Regarding the traditional two-stream method, despite the high AUROC score (0.7797), we can conclude from the confusion matrix (Figure 6) that the model does not discriminate against non-ASD well. This is discussed in detail in subsequent sections.

The confusion matrix (Figure 6) shows that doctors 1 (Figure 6A) and 2 (Figure 6B) are poor at discriminating ASD but relatively well at discriminating non-ASD. We considered the movement characteristics of non-ASD individuals to be relatively obvious (dropped head and hip osteoarthritis); however, the walking process of ASD individuals has not been clearly characterized. This makes discrimination based on doctors' observations more difficult. The traditional two-stream method shows good accuracy for ASD but poor accuracy for non-ASD. This indicates that the prediction of ASD was successful, but it was almost impossible to distinguish non-ASD. We assume that the OF is better at extracting motion information from the frames of the ASD but fails to capture spatial features (e.g., dropped head action). The proposed method has discriminatory capabilities for all the diseases. The scores of 0.8512 for TP and 0.6420 for TN indicate that the proposed method can simultaneously capture both spatial and temporal features during patient walking. This allows the network to effectively learn patterns and dependencies over time, making it suitable for tasks that involve analyzing sequential data such as videos.

An ablation study (Section 4.4) demonstrates that our preprocessing method (detection stage) is important for video diagnosis. The classification stage (3D CNN) is sensitive to unclear backgrounds, including tables and fans. In the classification stage, transfer learning is important for obtaining the final results. According to some studies (Karpathy et al., 2014; Kay et al., 2017), a large-scale dataset is required for training small-scale models. The highest prediction accuracy (0.7553) was obtained using a preprocessing method (detection stage) and transfer learning. Without the preprocessing method (detection stage), the final accuracy was approximately 0.5, indicating that the model accurately estimated the input data. Interestingly, the performance without the preprocessing method, even with transfer learning, was lower than that with training from scratch (0.4597 to 0.5134). We believe that transfer learning affects a complex background more than it does a clean background. The key advantage of the proposed method is its ability to effectively capture and analyze gait posture changes from video data. By utilizing a two-stage approach that integrates detection and classification analyses, we extracted gait posture features from video frames. The detection stage processes the detected body regions and extracts static features related to spinal deformities, whereas the classification stage analyzes frame information to capture the dynamic aspects of spinal motion during walking. The fusion of the two stages enhances the diagnostic accuracy of our method, making it more capable of capturing subtle movement changes in gait posture. Consequently, the proposed method provides a more comprehensive and accurate representation of gait patterns associated with ASD and other spinal disorders.

Model visualization (Section 4.5) with Figure 8 shows that, in the case of success, the model focuses more on the body parts of the patient, whereas in the case of failure, it focuses more on irrelevant parts (parts other than the patient). In terms of success, the model emphasized the positions of the head and feet. We believe that, during walking, the head and foot swings have the largest amount of movement; therefore, the classification stage learns more movement information from these parts. In the event of failure, the final prediction is incorrect, because the model does not focus well on the patient's body. One reason for this is that the classification stage focuses more on the background (the poster in Figure 8B and the curtain and wall in Figure 8D) and ignores the patient's features (body parts) when extracting the frames. By splitting the entire dataset into three parts, we analyzed the patient's results from the start to the end of walking. The metrics used are presented in Table 7. The accuracy for the entire dataset was 0.7553, compared with 0.7559 for the split dataset (mean of three different parts). The F1 score for the entire dataset was 0.7063, compared to 0.7079 for the split dataset. The AUROC for the entire dataset was 0.7864, compared with 0.7867, which was the mean AUROC for the split dataset. After a long period of walking, the metrics of Part B were higher than those of Parts A and C Therefore, we suspect that the patient's posture had changed since the beginning. The split dataset confusion matrix (Figure 7) indicates that the model can distinguish ASD equally (the difference in TP is approximately 0.01). However, the ability to distinguish non-ASD individuals varies. B (0.6603 PN) and C (0.6477 PN) parts are higher than Part A (0.6271 PN).

The proposed method for predicting the non-ASD category has a low performance (Figure 6D) because the non-ASD category involves other pathologies (DHS, LCS, HipOA). We believe that the classifier did not achieve representative characteristics of these pathologies. As this is the first study designed for ASD diagnosis, we paid more attention to the ASD category, and the treatment of non-ASD patients still has some shortcomings. In the future, we believe that the following ways can be used to solve the problem of insufficient categorization of non-ASD individuals:

- Based on the characteristics of different diseases, try to use techniques that emphasize the key part of the corresponding disease.
- Use additional algorithms to provide more information to enhance the key part importance.

Another shortcoming of this study is that the clinical assessment is not usually performed within 1 s of the patient walking. It could be solved by statistically comparing the doctor's observations and the performance of the proposed methods at different lengths of time to achieve fair diagnostic results of the doctor. However, we believe that 1 s, comparable to the time it takes a patient to walk to the doctor in a clinic examination room, would be more practical.

## 6 Conclusion

In summary, we propose a two-stage classification method for diagnosing ASD in patients using video format. We propose a framework that combines the preprocessing method (detection stage) and motion classification network (classification stage). In the detection stage, we introduce a preprocessing method to remove irrelevant information from videos and capture keyframes. During the classification stage, we introduced a ResNet style 3D CNN with video frame images to capture the spatiotemporal features (gait posture) of the patients while walking.

According to the experimental results, the proposed method outperformed the existing classification methods and the diagnosis of experienced doctors in terms of accuracy. Based on the ablation study, the preprocessing method (detection stage) appeared to be effective. Our transfer learning experiments suggest that the learned features are generic and can be generalized to other video classification tasks. Based on model visualization, our models focused on diagnosing illnesses, and classification failed because they did not focus on the patient.

In future studies, we plan to use a segmentation technique to capture clean body edges from frames. Model visualization shows that an irrelevant background is an influencing factor. Moreover, our viewpoint only calculates information from the character sides (left and right) and does not include information from the front or back. In future, we wish to use all four viewpoints (front, back, left, and right) for the character in disease analysis.

## Data availability statement

The original contributions presented in the study are included in the article/supplementary material, further inquiries can be directed to the corresponding author.

## Ethics statement

The studies involving humans were approved by the Ethics Committee of the University of Tsukuba Hospital (H30-087). The studies were conducted in accordance with the local legislation and institutional requirements. The participants provided their written informed consent to participate in this study.

## Author contributions

KC: Writing - original draft, Writing - review & editing, Data curation, Software, Methodology. TA: Writing - review & editing, Resources, Supervision. NI: Writing - review & editing, Supervision. KM: Writing - review & editing, Funding acquisition. KS: Writing - review & editing, Validation. TS: Writing - review & editing, Validation. HK: Writing - review & editing, Resources. MY: Writing - review & editing. YK: Writing - review & editing, Funding acquisition, Project administration.
